# Vitamin D_3_ and Monomethyl Fumarate Enhance Natural Killer Cell Lysis of Dendritic Cells and Ameliorate the Clinical Score in Mice Suffering from Experimental Autoimmune Encephalomyelitis

**DOI:** 10.3390/toxins7114730

**Published:** 2015-11-13

**Authors:** Zaidoon Al-Jaderi, Azzam A. Maghazachi

**Affiliations:** 1 Department of Physiology, Institute of Basic Medical Sciences, Faculty of Medicine, University of Oslo, POB 1103, Oslo N-0317, Norway; E-Mail: al-jaderi@medisin.uio.no; 2 College of Medicine, University of Sharjah, Al-Sharja 27272, United Arab Emirates

**Keywords:** natural killer cells, dendritic cells, experimental autoimmune encephalomyelitis, 1α,25-Dihydroxyvitamin D_3_, monomethyl fumarate, myelin proteolipid protein

## Abstract

Experimental autoimmune encephalomyelitis (EAE) is a CD4^+^ T cell mediated inflammatory demyelinating disease that is induced in mice by administration of peptides derived from myelin proteins. We developed EAE in SJL mice by administration of PLP_139–151_ peptide. The effect of treating these mice with 1α,25-Dihydroxyvitamin D_3_ (vitamin D_3_), or with monomethyl fumarate (MMF) was then examined. We observed that both vitamin D_3_ and MMF inhibited and/or prevented EAE in these mice. These findings were corroborated with isolating natural killer (NK) cells from vitamin D_3_-treated or MMF-treated EAE mice that lysed immature or mature dendritic cells. The results support and extend other findings indicating that an important mechanism of action for drugs used to treat multiple sclerosis (MS) is to enhance NK cell lysis of dendritic cells.

## 1. Introduction

Natural killer (NK) cells perform several important functions; among them the regulation of the adaptive immune response by secreting cytokines such as IFN-γ [1], shaping the innate immune system by interacting with dendritic cells [2], defending against viral infection [3], and lysing and destroying tumor cells [4]. NK cells express many receptors which can be either inhibitory or activating. Resting NK cells respond to dangers occurring at sites of injury. Here, they change their adhesion molecules and up-regulate the expression of chemokine receptors that aid them in extravasation into injured tissues. In the blood circulation, human NK cells can be classified into two major subsets; those that express CD56 and low if any CD16 (known as CD56^bright/+^), and those that express CD16 and low CD56 (known as CD56^dim/−^) [1,4]. The former subset comprises about 10% of total NK cells in the blood, whereas the latter subset comprises about 90%. CD56^bright/+^ cells are more regulatory secreting IFN-γ and other cytokines and chemokines, but are less cytolytic than CD56^dim/−^ cells which are cytolytic but secrete cytokines with less intensity than the former cells. These two subsets also differ in their chemokine receptor expression [5]. While human NK cells are defined as CD3^−^CD56^−or+^ [6], murine NK cells lack the CD56 marker, and express NK1.1^+^ [7], which identifies NK cells from C57Bl/6, SJL and other mice strains [8].

The consensus is that the activity and numbers of NK cells in autoimmune diseases are reduced [9]. However, the role of NK cells in multiple sclerosis (MS) is controversial with one school of thought suggesting that NK cells ameliorate the disease, whereas another school indicates that they exacerbate the disease [10]. We previously observed that glatiramer acetate (GA) a drug approved for treating MS patients, enhances the cytolysis of activated human NK cells against autologous and allogeneic human immature and mature monocyte-derived DCs [11]. These results indicate that this drug is capable of activating NK cells to lyse monocyte-derived DCs, a concept that may have vital implications. The fact that NK cells exposed to GA kill both immature and mature DCs may lead to the inability of these cells to present antigens to autoreactive T cells. We have translated these findings to mice with EAE, where administration of GA ameliorates the EAE clinical scores [12]. Supporting these findings, we monitored the effects of NK cells to lyse DCs in MS patients for a period of one year and observed that dosing with GA enhances NK cell lysis of autologous DCs [13].

Vitamin D_3_ deficiency increases the risk of MS, as increased latitude is also correlated with lower blood vitamin D_3_ levels. For instance, ecological studies showed the amount of exposure to sunlight was inversely correlated with the risk of MS, by both regional distribution and as an association with altitude, as well as individual exposure to sunlight [14]. Sunlight is the main source of human vitamin D_3_ through conversion of 7-dehydrocholesterol to previtamin D_3_ in the skin, and through further metabolic steps to active hormone 1,25-dihydroxyvitamin D_3_ [15]. Dietary vitamin D_3_ intake may reduce the risk of MS in spite of latitude-dependent deficiency, for instance in areas where higher amounts of vitamin D_3_-rich fish are consumed [16]. There is also an association with disease in the EAE model, as dosing vitamin D_3_ prevented the disease [17,18]. Definite effects of supplementing patients with vitamin D_3_ have not yet been shown, but some studies indicate that serum concentrations of vitamin D_3_ may affect disease severity. It was also observed that MS patients receiving vitamin D_3_ have less relapses than control groups [19]. Also, increased serum level of vitamin D_3_ in MS patients resulted in improved T regulatory (Treg) cell activity, corroborated with suppression of auto-reactive T cells and a switch from a Th1 to Th2 phenotype [20]. This was later supported by showing an increase in the proportion of IL-10-secreting T cells after supplementing MS patients with vitamin D_3_ [21]. In cuprizone-fed animals, supplementation with this vitamin protected these animals from demyelination associated with reduced microglia activation and macrophage infiltration [22]. We reported that vitamin D_3_ and its derivative calcipotriol enhance *in vitro* NK cell lysis of dendritic cells (DCs), suggesting that a possible mechanism of action for these drugs is via activating NK cells [23].

Dimethyl fumarate (DMF) a drug used to treat multiple sclerosis (MS) patients, and its metabolite monomethyl fumarate (MMF) have the ability to protect from MS by enhancing Nuclear-factor (erythroid-derived 2)-related factor-2 (Nrf2), leading to the induction of Nrf-2 anti-oxidative pathway responses, thereby exerting neuroprotective effect by Nrf-2 mediated protection in MS tissues [24]. In EAE, DMF ameliorated the clinical course in myelin oligodendrocyte glycoprotein (MOG)-induced EAE in C57BL/6 mice. In addition, DMF suppressed Th1 and Th17 cell differentiation, as well as expression of pro-inflammatory cytokines IFN-γ, TNF-α, and IL-17 [25,26]. It also promoted Th2 cells that produce IL-4, IL-5, and IL-10 [27]. Additionally, vitamin D_3_ impaired dendritic cells (DCs) maturation which leads to reducing antigen presentation for encephalitogenic CD4^+^ T cells [28], and subsequently protecting the mice from developing EAE [26]. Recently, we described that MMF augments primary human CD56^+^ NK cell lysis of K562 and RAJI tumor cells [29]. However, the effect of MMF in MS patients and the mouse model EAE has not been clearly defined. The present work compares the effects of vitamin D_3_ and MMF in mice with EAE.

**Figure 1:**
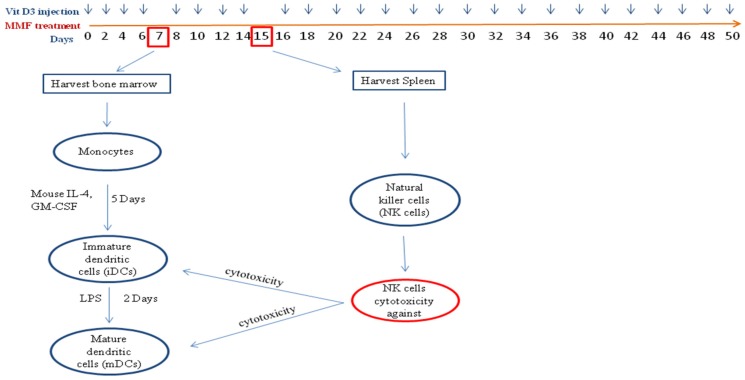
An overview of the study design. Mice were inoculated at day 0 with antigen and pertussis toxin (PTX). They were either left untreated or were treated with vitamin D_3_ at the indicated time points (blue arrows), or were fed with monomethyl fumarate (MMF) every day throughout the study. After 7 days, bone marrow was harvested to generate immature dendritic cells (iDCs) and mature dendritic cells (mDCs). At day 15 the spleens were isolated to generate natural killer (NK) cells which were used in the cytotoxicity assay.

## 2. Results

The protocol for the study design is shown in Figure 1. EAE was induced in SJL mice, and as a control normal mice were used. The first group of mice was left untreated, while the second was treated with vitamin D_3_, and the third group was fed with MMF, as shown in Figure 1.

### 2.1. Vitamin D_3_ or MMF Reduces the EAE Clinical Score

**Figure 2:**
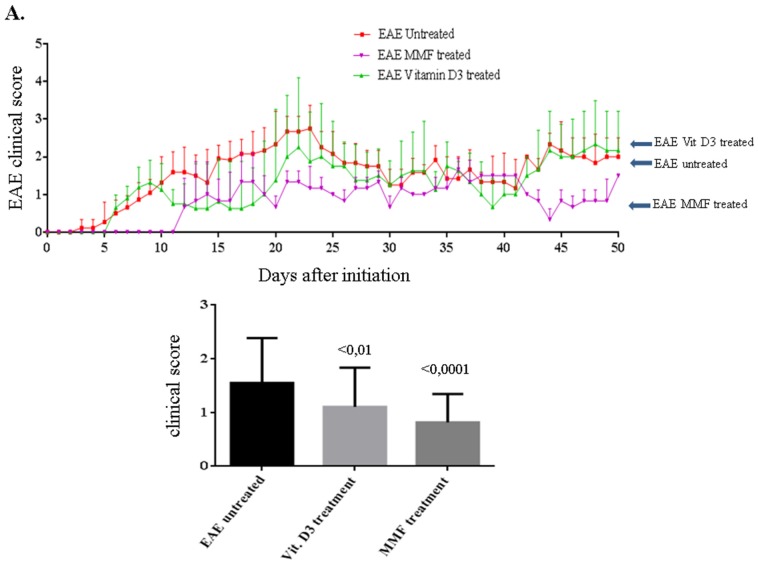

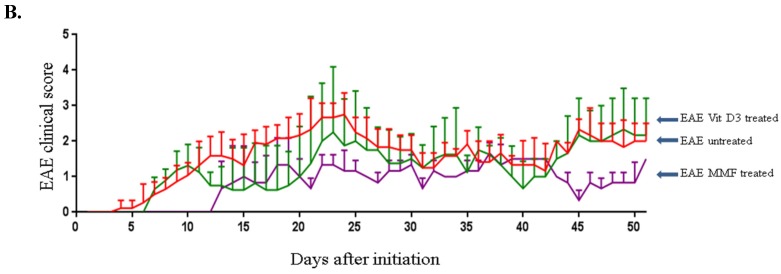
Comparison of the experimental autoimmune encephalomyelitis (EAE) clinical score among untreated mice (red line), mice treated with vitamin D_3_ (green line) or those fed MMF (pink line) for 50 days. The significant values were calculated using one way ANOVA followed by Sidak’s multiple comparison test during the entire period of the experiments (**A**). Results were also evaluated by area under curve analysis (**B**). Data were collected from 10 mice in each group at any time point.

First we sought to demonstrate if injecting the mice with vitamin D_3_ or feeding them with MMF might reduce the incidence of EAE. During the 50 days of measuring the EAE clinical score, it was observed that injecting vitamin D_3_ significantly reduced the EAE clinical score in these mice (*P* < 0.01, Figure 2). However, the best reduction in the EAE clinical score was observed in mice fed with MMF (*P* < 0.0001 as compared to EAE mice that were left untreated, Figure 2A). Similar outcome was observed when the data were evaluated by area under curve analysis (Figure 2B).

### 2.2. Vitamin D_3_ Induces NK Cell Lysis of Dendritic Cells

**Figure 3:**
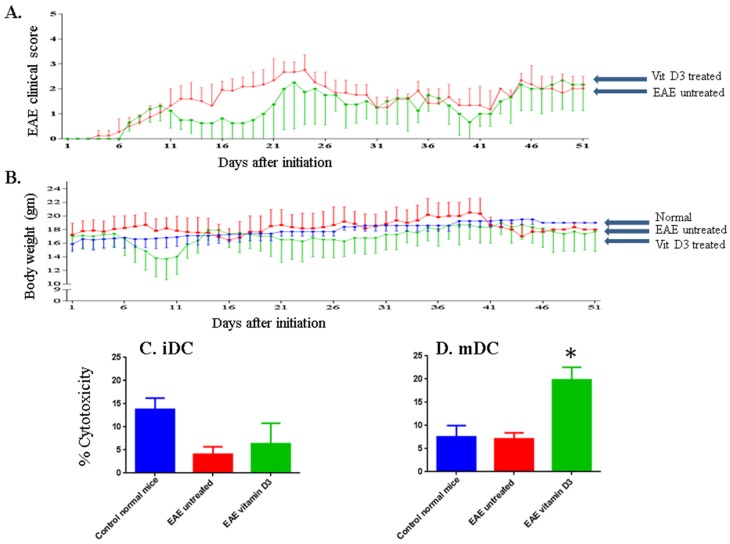
Vitamin D_3_ ameliorates the clinical score in mice with EAE and induces NK cell lysis of DCs. EAE clinical score in mice injected IP with vehicle (red line) or mice injected IP with vitamin D_3_ (green line) for 51 days (**A**). The body weight of untreated EAE mice (red line), vitamin D_3_ treated mice (green line), or normal mice where no disease was induced (blue line) was also examined (**B**). Mean ± SEM of 3 female mice ages 4–6 weeks from each group at each time point is shown. Vitamin D_3_ increases NK cell lysis of DCs. NK cells isolated from normal mice, EAE untreated mice or mice injected IP with vitamin D_3_, were examined for their ability to lyse iDCs (**C**) or mDCs (**D**). Immature DCs were generated by isolating bone marrow cells day 7 after initiation of the disease and then incubating them *in vitro* with murine GM-CSF plus IL-4 for 5 days (Figure 1). The cells were then incubated for an additional 2 days with LPS to generate mature DCs. E:T ratio is 50:1. Significant values were calculated using one-way ANOVA. * *P* < 0.001.

In this series of experiments, we sought to confirm the effect of injecting vitamin D_3_ on the development of EAE in SJL mice and correlated this effect with the ability of the drug to activate NK cells. As shown in Figure 3A, the disease developed in these mice after six days of initiation. In control mice, the trend of EAE clinical score increase was noted until day 23 after initiation and then subsided at day 30. In contrast, there was almost a complete abrogation of EAE in mice treated with vitamin D_3_ between 15–17 days of drug treatment. The disease rebounded in these mice after 22 days of treatment but did not reach the level of untreated mice until day 26 (Figure 3A). Further, we compared the body weight of normal mice to those mice suffering from EAE but were untreated with any drug, or in EAE mice treated with vitamin D_3_. Surprisingly, mice treated with vitamin D_3_ lost weight after 7–10 days of treatment but then restored their weight after about 12–18 days post treatment (Figure 3B). The reason behind weight loss in these mice is not clear. Similar weight loss was observed in another EAE mice model where vitamin D_3_ either alone or in combination with an antigen was used to treat these mice where EAE was induced with myelin oligodendrocyte glycoprotein [30].

We previously reported that one of the possible mechanisms of action for vitamin D_3_ is by enhancing *in vitro* NK cell lysis of immature (i) and mature (m) dendritic cells (DCs) [23]. To investigate whether similar activity might be functional in EAE mice, we isolated both NK cells and DCs from these mice, and then measured NK cell lysis against DCs. The phenotypes of isolated NK cells are shown in supplementary Figure S1, where most of the cells express NKp46, and to a lesser extent NKG2D and NK 1.1. In these experiments, NK cells and DCs were isolated from the same treated group. Hence, NK cells isolated from vitamin D_3_-treated mice were examined for their ability to kill DCs isolated from the same animals, and so on (see Figure 1). The premise for this is to examine the effect of the treatment on NK cell lysis, hence resembling what happens in treated individuals. Results in Figure 3C demonstrate that NK cells isolated from normal mice are capable of lysing iDCs, and that this effect was significantly reduced in EAE mice, suggesting that in these mice NK cell activity is inhibited. NK cell lysis of mDCs was minimal in normal mice. Consequently, it was not feasible to observe any effect of EAE disease on such activity. Treatment with either vitamin D_3_ significantly augmented NK cell lysis of mDCs in mice suffering from EAE (*P* < 0.001 as compared to untreated EAE mice, Figure 3D).

**Figure 4:**
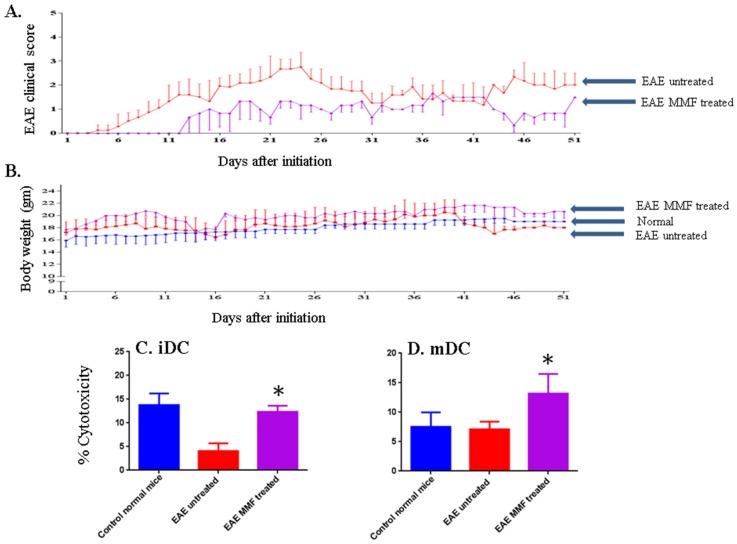
MMF reduces the EAE clinical score and enhances NK cell lysis of DCs. EAE clinical score in mice gavaged MMF (pink line), or control mice treated similarly to MMF-gavaged mice, except that vehicle was used instead of MMF (red line) (**A**). Body weights of mice treated with MMF (pink line), untreated EAE mice (red lines), or normal mice where no disease was induced (blue line) are shown (**B**). MMF treatment increases NK cell lysis of iDCs (**C**) or mDCs (**D**). E:T ratio is 50:1. Significant values were calculated using one-way ANOVA. * *P* < 0.05.

### 2.3. MMF Reduces the EAE Clinical Score Corroborated with Enhancing NK Cell Activity

The effects of treatment with MMF on EAE clinical score and body weight are shown in Figure 3. The results confirmed those observed earlier using 10 mice from each group (see Figure 2). The disease started to develop after about 6 days of initiation in untreated mice. However, there was a delay in EAE clinical score until day 12 in mice gavaged with MMF. Further, there was a continuous reduction in the EAE clinical score in mice fed MMF throughout the 50 days of measuring the disease score in these mice (Figure 4A). Surprisingly, mice fed MMF gained weight throughout the study hence, the weight of these mice was much higher than normal mice (Figure 4B). In contrast, mice suffering from EAE which were not treated with the drug lost weight compared to normal mice or mice treated with MMF (Figure 4B). To correlate these findings with activating NK cell lysis of DCs, we isolated NK cells from MMF fed mice and from untreated mice as well as from normal mice. A clear and significant enhancement of NK cell lysis against iDCs was observed in mice fed with MMF (*P* < 0.05, Figure 4C). Similarly, treatment with MMF significantly augmented NK cell lysis of mDCs in mice suffering from EAE (*P* < 0.05 as compared to untreated EAE mice, Figure 4D).

**Figure 5:**
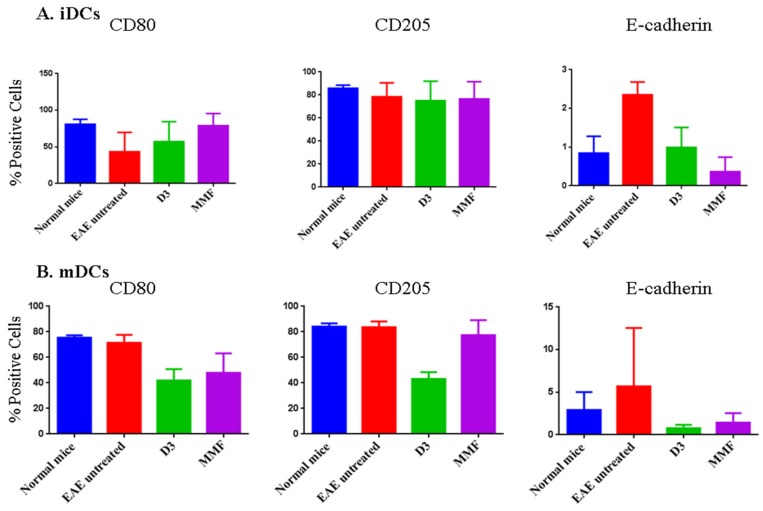
Effect of treating mice with vitamin D3 or MMF on the expression of various molecules on the surface of DCs. iDCs or mDCs were isolated from normal untreated mice, EAE mice, EAE mice injected IP with vitamin D3 or mice orally fed MMF. These cells were labeled with antibodies towards CD80, CD205 or E-cadherin and then examined in the flow cytometry. Mean ± SEM of three experiments except for E-cadherin in mDCs where only two experiments were performed.

### 2.4. Effects of Vitamin D_3_ and MMF on Dendritic Cell Phenotypic Expression

It was previously reported that MMF affects DCs differentiation [31], or monocyte polarization [32]. To investigate whether enhancing NK cell lysis of DCs might be due to changes in DCs phenotypic expression, we examined the expression of various surface molecules in iDCs and mDCs isolated from normal mice, mice with EAE, mice with EAE injected with vitamin D_3_, or mice with EAE fed MMF. We chose three molecules important for DCs maturation; these included the co-stimulatory molecule CD80, recognition and adhesion molecules CD205 and E-cadherin [33]. We observed no significant effects on the expression of CD80 molecule in iDCs (Figure 5A), or mDCs (Figure 5B), after treating the mice with vitamin D_3_ or MMF. No effect on the expression of CD205 was seen in iDCs (Figure 5A), but vitamin D_3_ treatment reduced the expression of this molecule on the surface of mDCs (Figure 5B). E-cadherin expression was up-regulated in EAE mice, and that both vitamin D_3_ and MMF reversed this up-regulation on iDCs (Figure 5A). However, no effect of treatments on E-cadherin was observed in mDCs (Figure 5B).

## 3. Discussion

We previously reported that drugs used to treat multiple sclerosis have one mechanism of action in common, in addition to their other functions. NK cells incubated *in vitro* with GA robustly killed iDCs and mDCs [11]. Similarly, NK cells collected from EAE mice or from MS patients dosed with GA killed both types of DCs [12,13]. Further studies showed that FTY720 (finglimod), and vitamin D_3_ also enhanced *in vitro* NK cell lysis of DCs [23].

The ability of vitamin D_3_ or another candidate drug for MS, namely MMF to enhance NK cells has not been examined *in vivo*. Several studies showed that vitamin D_3_ ameliorated EAE and improved the clinical score in EAE mice [34]. One mechanism of action reported for this vitamin is inhibiting Th1 and Th17 responses as well as inflammatory cytokines secreted by these cells [35,36,37,38,39]. Consequently, it induced the shift of the immune system towards a more anti-inflammatory type 2 response [40]. Lower levels of IFN-γ, IL-17, IL-6, and IL-10 were reported in EAE mice treated with vitamin D_3_ [30]. Vitamin D_3_ was also shown to improve the function of Treg cells in relapsing remitting MS patients [20]. In addition, 1,25 (OH)_2_ vitamin D_3_ increased Fas expression on melanoma cells making them susceptible to NK cell induced-apoptosis [41]. High circulating levels of vitamin D_3_ binding proteins in MS patient may result in more relapses [42].

Similar to these mentioned reports, we describe here that vitamin D_3_ ameliorates EAE. Mice treated with this vitamin lost weight early after treatment but this could be due to calcemia that could be effective during early days after treatment. In another EAE mouse model, it was reported that treatment with vitamin D_3_ also resulted in weight losses [30]. What is important is the ability of vitamin D_3_ to enhance NK cell lysis of mDCs. It could therefore be suggested that one of the mechanisms of vitamin D_3_ activity is impeding antigen presentation to autoreactive T cells.

On the other hand, dimethyl fumarate (DMF) is currently being used to treat patients with MS, under the name Tecfidera (Biogen). In EAE, DMF exerts clinical effects by reducing macrophage-induced inflammation in the spinal cord [43]. MMF is the metabolite of DMF, where one methyl group is lost due to hydrolysis by esterases in the intestine following oral administration. It was observed that free DMF could not be detected in plasma of the portal vein blood after oral application of DMF in rats, mainly due to its conversion to MMF, and the formation of adducts with glutathione [44]. For this reason it was suggested that MMF might be the active molecule of DMF, although this is not proven yet.

Though the effects of DMF in EAE or MS patients have been studied, those related to the activity of MMF are scarce. Scannevin *et al.* [44] demonstrated that MMF increased the cellular level of glutathione in astrocytes. This is corroborated with alleviating some of mitochondrial dysfunctions. The authors also reported an increase in the anti-apoptotic molecule nuclear factor (erythroid-derived)-2 (Nrf2). These results were supported by others showing that MMF protected cultured neurons and astrocytes from hydrogen peroxide-induced cell death [24]. It was also reported that MMF is an agonist of the hydroxycarboxylic acid receptor 2 (HCA2, a G protein coupled receptor also known as GPR109A) [45] and that HCA2 mediates the therapeutic effects of DMF or MMF in the mouse model of EAE [46]. However, whether MMF *per se* has any effect in mice suffering from EAE has not been clearly shown. Our results demonstrate that this molecule reduces the clinical score in mice suffering from EAE. In fact, the activity of MMF is superior to vitamin D_3_. The effect of MMF is also correlated with the ability of this drug to enhance NK cell lysis of DCs. Hence, NK cells isolated from mice suffering from EAE significantly killed iDCs and mDCs isolated from the same mice. These results suggest that one important mechanism of action in reducing EAE clinical score by MMF is via removing those DCs responsible for activating T cells which cause damages to the myelin sheath during the course of the disease. It was previously reported that MMF affects the differentiation and polarization of DCs [31,32]. We observed some effect of vitamin D_3_ on the expression of CD205 in mDCs, and that vitamin D_3_ and MMF affect E-cadherin expressed in iDCs. However, there was no consistency among the effects of these drugs on iDCs and mDCs to justify enhancing NK cell lysis of both types of DCs. Consequently, we conclude that the effects of these drugs are plausibly exerted on NK cells during treatment. In summary, our findings are the first to demonstrate that one function of vitamin D_3_ or MMF (not yet used for therapy) is perhaps due to activating NK cells to lyse DCs.

## 4. Experimental Sections

### 4.1. Ethical Statement

All animal studies and procedures were approved by Norwegian Animal Research Authority (FOTS) and Department of Comparative Medicine, University of Oslo. Female SJL/J mice at four to six weeks age were purchased from Jackson Laboratory. Mice were kept under pathogen-free conditions at the University of Oslo.

### 4.2. Induction of EAE in SJL Mice

Female SJL/J (H-2ˢ) mice ages 4–6 weeks old were immunized subcutaneously (SC) with 200 μg of PLP_139–151_ peptide purchased from ABBIOTEC (San Diego, CA, USA) emulsified in complete Freund’s adjuvant (CFA) containing 1 mg *Mycobacterium tuberculosis* (Sigma-Aldrich, Oslo, Norway), at four sites in the right and left flanks. Following each injection, 200 ng of *Bordetella pertussis* toxin (EMD chemicals, Darmstadt, Germany) was injected intraperitoneal (IP) after 0 and 48 h after immunization with the peptide. The animals were independently observed and monitored daily, and the EAE clinical score was measured according to the following scoring scheme. 0 = no clinical disease, 1 = tail flaccidity, 2 = hind limb weakness, 3 = hind limb paralysis, 4 = forelimb paralysis, and 5 = moribund or death.

### 4.3. Treatment of Mice and Isolation of NK Cells and DCs

SJL/J mice were divided into several groups and each group consisted of 10 mice each. The first group was left as control without induction of EAE, the second group where EAE was induced was left without treatment but injected IP with vehicle, and the other group was gavaged with vehicle control. Treated mice include those injected IP with 100 ng 1α,25-Dihydroxyvitamin D_3_ (Sigma-Aldrich), the active form of vitamin D_3_ every other day, or mice orally gavaged every day with 1 mg MMF (Sigma-Aldrich). On day 7, bone marrow (BM) cells were flushed from the tibia and femur of mice, and the monocytes were isolated using EasySep mouse monocyte Enrichment kit (STEMCELL Technologies SARL, Grenoble, France). For generation of iDCs, monocytes were incubated at 2 × 10^6^ cells/mL supplemented with 25 ng/mL recombinant murine GM-CSF and 6 ng/mL recombinant murine IL-4 (Pepro Tech Ltd., London, UK), in culture dishes. Mature dendritic cells (mDCs) were generated by adding 1 μg/mL lipopolysaccharide (LPS) (Sigma-Aldrich). At day 15 post immunization, 5 mice were euthanized with CO_2_ and spleens were isolated. NK cells were purified from splenocytes using EasySep mouse NK Enrichment kit from STEMCELL Technologies SARL. These NK cells were used to lyse iDCs or mDCs.

### 4.4. NK Cells Cytotoxicity Assay

For NK cell lysis of immature and mature dendritic derived monocytes cells, target cells were incubated at 1 × 10^6^ cells/mL with 5 μg/mL calcein-AM (Teflabs, Austin, TX, USA) for 1 h at 37 °C in a 5% CO_2_. Target cells were washed twice and plated at 10,000 cells/well with NK cells into 69-well flat bottom plates at 50:1 E:T ratio in triplicate. The plates were spun down at 500 rpm for 5 min and incubated for 4 h at 37 °C in 5% CO_2_. To obtain total killing, target cells were incubated with 0.5% Triton-X (Sigma-Aldrich) for 30 min, whereas total viability was obtained by incubating the cells with medium only. The plates were centrifuged for 8 min and medium was replaced with Dulbecco’s Phosphate Buffered Saline (DPBS) without Ca and Mg (Sigma-Aldrich). The fluorescence intensity of the calcein-AM loaded target cells was measured with BioTek FLX 800 plate reader (Bio-Tek Instruments Inc., Winooski, VT, USA), using 485/528 nm fluorescence filters. The percentage of cytotoxicity was calculated according to the following formula: % Viability = Fluorescence units (FU) of targets incubated with NK cells (experimental), minus FU of targets incubated with Triton-X (total lysis), divided by FU of targets incubated in media only (total viability), minus FU of targets incubated with Triton-X (total lysis). Percent cytotoxicity was then calculated as 100% minus % viability as described [24].

### 4.5. Flow Cytometry Analysis (FACS)

NK cells (3 × 10^5^) were washed, suspended in a FACS-buffer (PBS without Ca, Mg, 2%FBS, and 10 mM NaN_3_), and labeled in the dark for 45 min at 4 °C with FITC-conjugated rat IgG2a, κ isotype control, APC-conjugated rat IgG, κ isotype control, FITC-conjugated rat anti mouse CD335 (NKp46), APC-conjugated rat anti-mouse NKG2D (CD314) (All antibodies were obtained from BD Pharmingen, San Diego, CA, USA). PE-conjugated mouse anti-NK1.1 (Southern Biotech, Birmingham, AL, USA), and PE-conjugated mouse IgG2a isotype control (ImmunoTools, Friesoythe, Germany) were used to stain NK cells. The cells were washed twice, medium was replaced with PBS and the cells analyzed in a flow cytometer (FACS Calibur, Beckton Dickinson Biosciences, San Jose, CA, USA). Gating was done according to the isotype controls, and the analysis was performed using FlowJo (Flow cytometry analysis software, Ashland, OR, USA).

To label dendritic cells, iDCs or mDCs cells were incubated with FITC-conjugated hamster anti-mouse CD80 (B7-1), or FITC-conjugated hamster IgG isotype control (eBioscience, Inc., San Diego, CA, USA). They were also labeled with fluorescein-conjugated rat anti-mouse E-Cadherin, FITC-conjugated rat IgG2A isotype control, PE-conjugated rat anti-mouse DEC-205, or PE-conjugated rat IgG2B isotype control (R&D Systems, Minneapolis, MN, USA). Labeled cells were washed twice, media replaced with PBS, and then analyzed in flow cytometry.

### 4.6. Statistical Analysis

Significant values (*P* < 0.05) were calculated using the student *t*-test, or one way ANOVA followed by Sidak’s test analysis calculated by Graphpad Prism 6 program (San Diego, CA, USA). Area under curve analysis was performed using the Graphpad Prism 6 program.

## 5. Conclusions

Our results are one of the first to show that MMF ameliorates EAE clinical scores in mice. The effect of MMF is comparable or superior to the effect of vitamin D_3_. Both drugs activate NK cells to lyse immature and mature DCs. This effect is similar to the function of other drugs used to treat MS patients such as glatiramer acetate, fingolimod and natalizumab. The fact that NK cells exposed to these drugs kill both immature and mature DCs may result in the inability of DCs to present antigens to autoreactive T cells. Finally, it is safe to conclude that most if not all drugs used to treat MS have a common function, *i.e.*, enhancing NK cell lysis of DCs. Therefore, we suggest that this method can be used as a screening tool to test any new drug before more efforts and money are put into investigating newly developed MS drugs.

## Supplementary Files

Supplementary File 1
