# Supplementary figures and images for: Vitamin D3 and Monomethyl Fumarate Enhance Natural Killer Cell Lysis of Dendritic Cells and Ameliorate the Clinical Score in Mice Suffering from Experimental Autoimmune Encephalomyelitis
Source: Toxins (Basel). 2015 Nov 13;7(11):4730–44. doi: 10.3390/toxins7114730 (PMC4663530; doi:10.3390/toxins7114730)

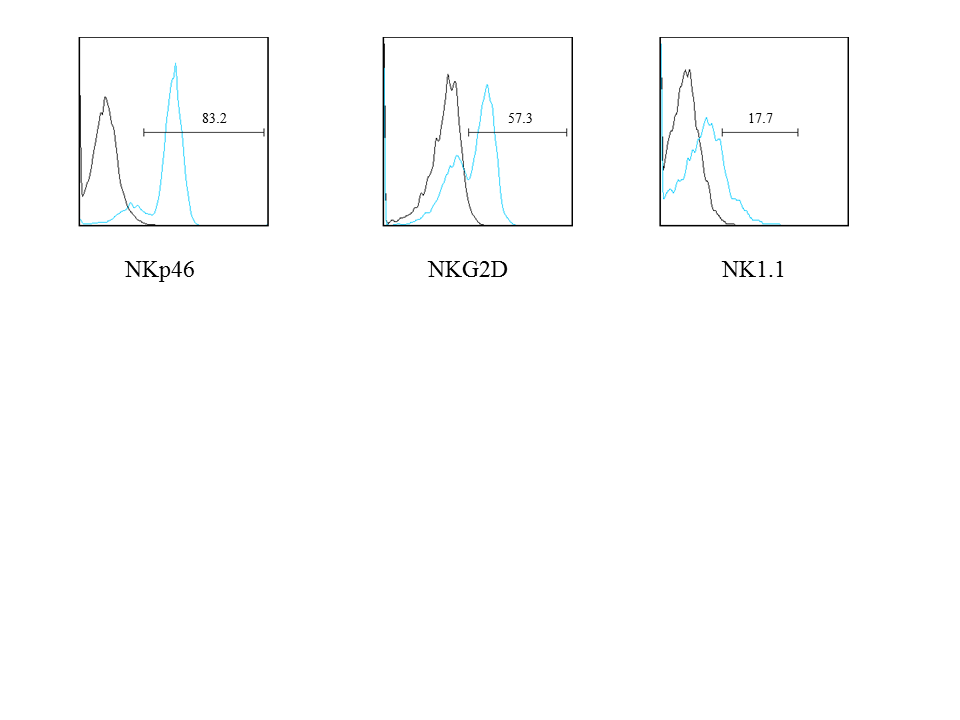

Supplement: Supplementary File 1 [file toxins-07-04730-s001.tiff]
